# Zinc deficiency and low enterocyte zinc transporter expression in human patients with autism related mutations in SHANK3

**DOI:** 10.1038/srep45190

**Published:** 2017-03-27

**Authors:** Stefanie Pfaender, Ann Katrin Sauer, Simone Hagmeyer, Katharina Mangus, Leonhard Linta, Stefan Liebau, Juergen Bockmann, Guillaume Huguet, Thomas Bourgeron, Tobias M. Boeckers, Andreas M. Grabrucker

**Affiliations:** 1 Institute for Anatomy and Cell Biology, Ulm University, 89081 Ulm, Germany; 2 WG Molecular Analysis of Synaptopathies, Neurology Dept., Neurocenter of Ulm University, 89081 Ulm, Germany; 3 Institute of Neuroanatomy, Eberhard Karls University Tübingen, 72074 Tübingen, Germany; 4 Institut Pasteur, Human Genetics and Cognitive Functions Unit, 75015 Paris, France; 5 CNRS UMR 3571: Genes, Synapses and Cognition, Institut Pasteur, 75015 Paris, France; 6 University Paris Diderot, Sorbonne Paris Cité, Human Genetics and Cognitive Functions, 75013 Paris, France; 7 FondaMental Foundation, 94010 Créteil, France

## Abstract

Phelan McDermid Syndrome (PMDS) is a genetic disorder characterized by features of Autism spectrum disorders. Similar to reports of Zn deficiency in autistic children, we have previously reported high incidence of Zn deficiency in PMDS. However, the underlying mechanisms are currently not well understood. Here, using inductively coupled plasma mass-spectrometry to measure the concentration of Zinc (Zn) and Copper (Cu) in hair samples from individuals with PMDS with 22q13.3 deletion including *SHANK3* (SH3 and multiple ankyrin repeat domains 3), we report a high rate of abnormally low Zn/Cu ratios. To investigate possible underlying mechanisms, we generated enterocytes from PMDS patient-derived induced pluripotent stem cells and used Caco-2 cells with knockdown of *SHANK3*. We detected decreased expression of Zn uptake transporters *ZIP2* and *ZIP4* on mRNA and protein level correlating with *SHANK3* expression levels, and found reduced levels of ZIP4 protein co-localizing with SHANK3 at the plasma membrane. We demonstrated that especially ZIP4 exists in a complex with SHANK3. Furthermore, we performed immunohistochemistry on gut sections from *Shank3αβ* knockout mice and confirmed a link between enterocytic SHANK3, ZIP2 and ZIP4. We conclude that apart from its well-known role in the CNS, SHANK3 might play a specific role in the GI tract.

The Phelan McDermid Syndrome (PMDS/22q13.3 deletion syndrome) is a rare genetic disorder characterized by symptoms of the autism spectrum that go along with mental retardation, muscular hypotonia, and severely delayed language development. Additionally, patients can show minor facial dysmorphisms, decreased perception of pain, and might suffer from seizures, ADD/ADHD (Attention Deficit Hyperactivity Disorder) and gastrointestinal (GI) problems^1^,^2^,^3^. PMDS is caused by a heterozygous deletion of the 22q13.3 region including *SHANK3* (SH3 and multiple ankyrin repeat domains 3, also known as proline-rich synapse-associated protein 2 (ProSAP2)). SHANK3 is a scaffolding protein of the postsynaptic density (PSD) of excitatory synapses^4^,^5^,^6^ known to be closely associated with autism spectrum disorders (ASD)^7^,^8^,^9^,^10^,^11^. *De novo* and inherited mutations in *SHANK3* have been found in individuals with ASD, and are present in about 0.69% of patients with ASD^11^. The protein has important functions at the synapse tethering receptors to the PSD, recruiting signaling complexes, and modulating actin polymerization, which underlines the focus on the synaptic site as central factor in the etiology or pathophysiology of ASD.

Heterozygous loss of *SHANK3* is considered to be the main cause for the neurological phenotype in PMDS since patients with small deletions or point mutations in the *SHANK3* gene often develop a phenotype close to that of PMDS including intellectual and speech impairment, hypotonia and ASD^1^,^12^,^13^,^14^. A clear association of deletion size and phenotype, however, could only be demonstrated for some features like developmental and language delay as well as hypotonia for larger deletions, and ASD for smaller deletions^15^,^16^. Thus, the genotype-phenotype correlation in PMDS and SHANK3 mutations is quite complicated. While it seems that many mutations in SHANK3 lead to the clinical picture of PMDS, not all of them do. On the other hand, deletion of SHANK3 seems to be critical for the development of PMDS, but features of PMDS might be modified by other deleted genes.

Similar to many reports of Zn deficiency or decreased Zn/Cu (increased Cu/Zn) ratio in autistic children^17^,^18^,^19^,^20^,^21^,^22^,^23^,^24^, we have previously reported a high incidence rate of Zn deficiency in blood samples of PMDS patients that was associated with the occurrence of seizures, ADHD or other attention and hyperactivity issues, and signs of immunodeficiency^25^.

Zn has a central role for GI development^26^, brain development and for a functional immune system. In humans, Zn deficiency has additionally been associated with several neuropsychiatric diseases such as depression and autism^27^,^28^,^29^,^30^,^31^. However, the pathophysiological correlations between Zn, Cu and ASD or PMDS are, especially on a molecular level, currently not well understood. Recent research indicates a link between the dysfunctions associated with ASD, Zn deficiency and GI problems^26^. Children with ASD frequently suffer from GI problems such as diarrhea, constipation, bloating, abdominal pain, and gastroesophageal reflux^26^.

In animal models, severe prenatal Zn deficiency is associated with teratogenic effects^32^, while mild or late prenatal and early postnatal Zn deficiency result in much less defined clinical pictures. Here, Zn-related dysfunctions lead to behavioral abnormalities, such as reduced memory and learning capacity, increased anxiety, and autism-like behavior^33^,^34^. It was also shown that a low Zn level may lead to Cu toxicity^28^, which can cause neurological deficits in children.

Zn homeostasis is regulated by many different proteins involved in absorption, intracellular buffering, trafficking and excretion, such as transporters of the ZIP (Zrt- and Irt-like proteins (SLC39A)) and ZnT (Zinc Transporter (SLC30A)) families, and metallothioneins (MTs). The main site of absorption of Zn is the small intestine, where Zn is absorbed by enterocytes. Of at least 10 ZnT and 15 ZIP transporters in human cells, several are expressed in enterocytes and mediate the cellular uptake (mostly ZIP) and removal (mostly ZnT) of Zn^29^. In particular, ZIP1 (SLC39A1), ZIP2 (SLC39A2), and ZIP4 (SLC39A4) localize to the apical membrane of enterocytes and are strongly involved in Zn absorption^35^. In times of decreased dietary Zn intake, in mice, ZIP4 is up-regulated underlining its function in the homeostatic control of Zn levels. Additionally, mutations in *ZIP4* are known to cause *Acrodermatitis enteropathica*, a rare autosomal recessive disorder of impaired Zn uptake in humans^36^.

To elucidate mechanistic insights on the dysregulation of heavy metal ions in ASD, we concentrated on one of the most prominent forms of syndromic ASD – PMDS. To that end we investigated Zn levels in PMDS individuals with a heterozygous 22q13.3 deletion including *SHANK3.* We found a high incidence rate of Zn deficiency also in this group of patients, with a syndromic form of ASD. Thus, we analyzed enterocytes differentiated from patient derived induced pluripotent stem cells (iPSC). Interestingly, we found that SHANK3 is expressed both in human enterocytes and in gut epithelium of mice. In a screening approach with 27 trace metal homeostasis genes, the Zn transporters ZIP2 and ZIP4 were identified reduced in enterocytes differentiated from PMDS iPSC. Reduced expression of ZIP2 and ZIP4, was confirmed on both mRNA and protein level. In addition, a decreased amount of ZIP4 protein was detected in SHANK3 protein complexes at the cell surface. Endogenous protein levels of SHANK3 seem to regulate the Zn transporter levels, as overexpression and knockdown experiments in cell lines demonstrated. Accordingly, low enterocytic SHANK3 levels result in diminished Zn transporter levels that could eventually explain reduced Zn concentrations in tissues and organs.

## Results

### Increased incidence of Zn deficiency and abnormal Zn/Cu ratio in individuals with PMDS

**Figure 1:**
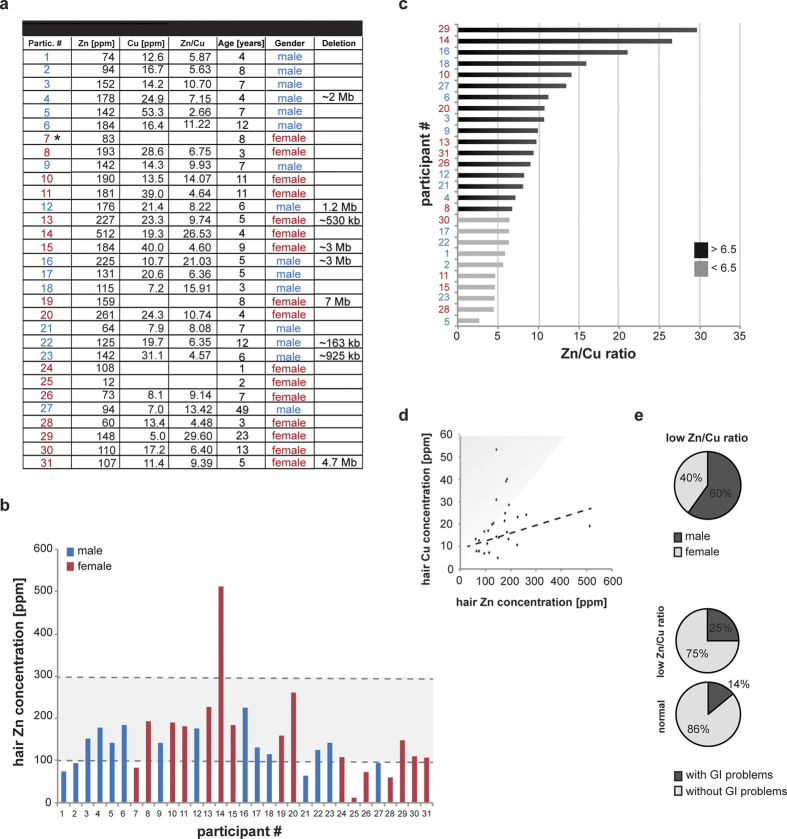
Zinc (Zn) and Copper (Cu) concentrations in the hair of individuals diagnosed with PMDS. (**a–d**) Hair samples were collected from 31 participants diagnosed with PMDS (15 males and 16 females) between 1 and 49 years of age (mean 8.3 years). **(a)** Concentrations of Zn and Cu were determined using ICP-MS and are shown in ppm. The patient marked with * was used for the generation of iPSC. **(b)** Eight participants were identified with Zn levels below the normal range (<95 ppm) and four showed abnormal high levels of Cu (>30 ppm). **(c)** The Zn/Cu ratio was calculated. A Zn/Cu ratio below 6.5 is considered below the normal range and was found in ten participants (32.3%). The grey zone in panel **(d)** marks the 6.5 cut-off limit. The dotted line corresponds to the average Zn/Cu ratio of patients without altered Zn/Cu ratio. **(e)** Of the participants with low Zn/Cu ratio, 60% were males. The occurrence of a low Zn/Cu ratio was not associated with gastro-intestinal (GI) problems given that 75% of participants with low Zn/Cu value did not report GI problems, similar to 86% of participants with normal Zn/Cu ratio.

Determination of Zn levels in blood revealed an increased incidence rate of Zn deficiency in PMDS^25^. To substantiate our findings, we assessed Zn levels in hair samples via inductively coupled plasma mass spectrometry (ICP-MS). Metal levels in hair samples represent a mean concentration of approx. 2 months^37^. Zn and Cu concentrations of 31 individuals (15 males and 16 females) between 1 and 49 years of age diagnosed with PMDS were measured (Fig. 1a). Only participants with a genetically confirmed diagnosis of PMDS and deletions in 22q13 affecting the *SHANK3* gene were included in the study. Minimum Zn concentrations were based on the levels necessary for individuals aged 1–12 that are less compared to those of adults. Eight participants (25.8%) were identified with Zn deficiency (Fig. 1a,b) and four showed abnormal high levels of Cu (Fig. 1a). Among a control group with age range and gender distribution similar to the group of participants with PMDS, an incidence rate of only 8.7% was found (Supplementary Fig. 1a). This results in a 197% increased incidence rate of Zn deficiency in PMDS patients compared to healthy controls. Based on the values obtained, the Zn/Cu ratio was calculated, that is a recommended biomarker for ASD patients^18^,^21^,^22^. A Zn/Cu ratio below 6.5, the lower limit considered a normal range^38^, was found in ten participants (Fig. 1c,d). Among the participants with low Zn/Cu ratios, the majority were males (Fig. 1e). The occurrence of Zn deficiency alone (data not shown) or a low Zn/Cu ratio was not associated with acute gastro-intestinal problems (Fig. 1e) in PMDS patients. GI abnormalities were assessed categorized into “frequent GI infections”, “constipation”, “diarrhea”, “flatulence”, and “other GI problems (not specified)”. All sub-categories were represented to similar extent within the PMDS group. No GI problems were reported in the control group.

The fraction of individuals with low Zn/Cu ratio seems to slightly decrease with age (Supplementary Fig. 1b). Participants with low Zn/Cu ratio were frequently found to show symptoms of ADD/ADHD (Supplementary Fig. 1c,d) which is in line with recent findings of an abnormal Zn/Cu ratio in ADHD^28^,^39^.

We were able to document the trace metal profile of one PMDS patient who displayed low Zn levels and that was reported to have undergone Zn supplementation with 10 mg Zn per day for 3 weeks (Supplementary Fig. 1e,f) (Zn supplementation was performed independently of this study by the patient’s legal guardians). The results show a strong increase in Zn content after a period of Zn supplementation with only small effect on other trace elements such as Cu, Pb, Hg, Fe, Ca, and Mg (Supplementary Fig. 1e). Accordingly, the Zn/Cu ratio showed a strong increase after Zn supplementation (Supplementary Fig. 1f) that went back to pretreatment values (Zn deficiency) after the discontinuation of the supplementation (Supplementary Fig. 1e,f).

### Abnormal enterocyte Zn transporter expression in hiPSCs from PMDS patients

**Figure 2:**
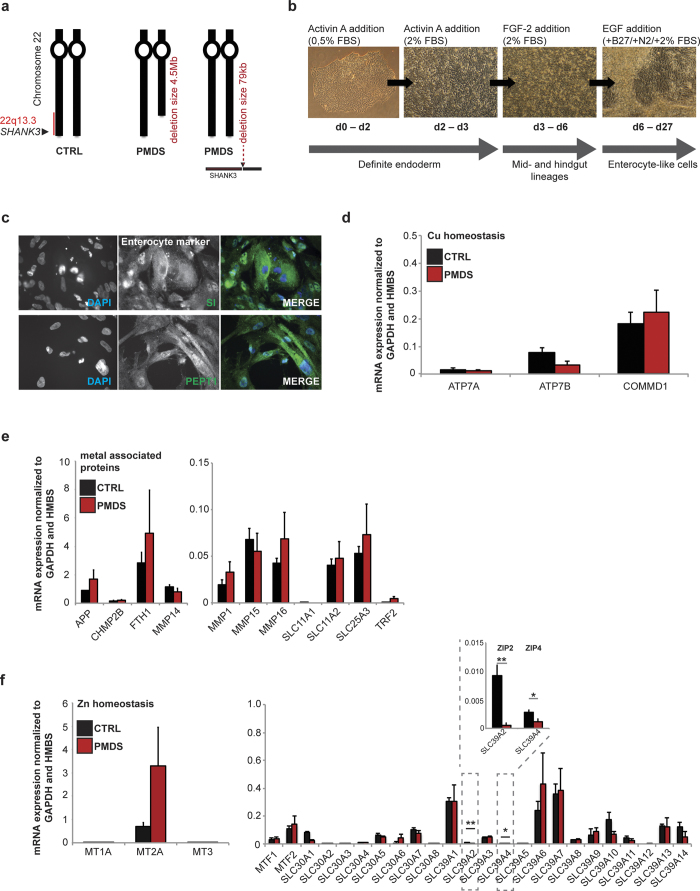
Expression-analysis of trace metal homeostasis genes in enterocytes derived from iPS cells from healthy and PMDS individuals. **(a)** Enterocytes were generated from 2 healthy controls and 2 PMDS patients. To that end, iPS cells were established from keratinocytes and differentiated into enterocytes (**b**). **(c)** Successful differentiation was controlled by expression analysis of the enterocyte marker proteins Sucrase-Isomaltase (SI) and Peptidase 1 (SLC15A1). (**d–f**) Quantification of mRNA expression in lysate obtained from enterocytes. Results show data pooled from 2 controls and 2 PMDS patients. Experiments were performed in triplicates to analyze genes involved in (**d**) copper homeostasis, (**e**) homeostasis of iron and other divalent metal ions, and (**f**) zinc homeostasis. **(d)** No significant alterations were seen in ATPase Cu^2+^-transporting beta polypeptide (*ATP7B*), ATPase Cu^2+^-transporting alpha polypeptide (*ATP7A*), and copper metabolism (*MURR1*) domain containing 1 (*COMMD1*). **(e)** Zn binding proteins such as Matrix metalloproteinases (*MMP*s), Amyloid precursor protein (*APP*), chromatin modifying protein 2B (*CHMP2B*), divalent metal ion transporters (*DMT1*/*SLC11A1, DMT2*/*SLC11A2*, and *SLC25A3*) and genes involved in iron homeostasis such as Ferritin heavy chain (*FTH1*) and transferrin receptor 2 (*TRF2*) showed no significant changes. **(f)** Analyzing important Zn homeostasis genes of the metallothionein family (*MT1A, MT2A, MT3*), metal-responsive transcription factor family (*MTF1, MTF2*), zinc transporter family (*ZnT*/*SLC30A*) and Zrt- and Irt-like proteins (*ZIP*/*SLC39A*), a trend towards increased levels of *MT2A* and a significant reduction in *SLC39A2 (ZIP2*) and *SLC39A4 (ZIP4*) (enlarged panel) was observed. (d-f) (t-test, n = 3 technical replicates from 2 cell lines per group; *ZIP2* (CTRL vs. PMDS) p = 0.0033; *ZIP4* (CTRL vs. PMDS) p = 0.0293).

To investigate whether abnormal Zn/Cu levels result from impaired absorption in the GI tract, we generated enterocytes derived from iPS cells^40^ from two healthy and two PMDS individuals (Fig. 2a,b; Supplementary Fig. 2) including one patient with measured Zn deficiency, while Zn status of the other patient was unknown.

**Figure 3:**
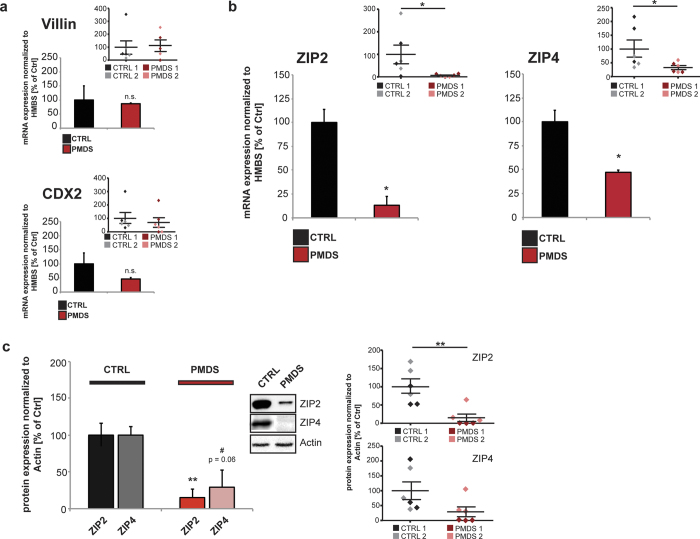
Analysis of ZIP2, ZIP4 and Zn biology in enterocytes from healthy and PMDS individuals. (**a,b**) Quantification of mRNA expression in lysate obtained from enterocytes. Results show data pooled from 2 controls and 2 PMDS patients. Smaller inserts (top right of graphs) show individual data points for each measurement and cell line per group. (**a**) Villin and CDX2, markers for enterocyte differentiation verify that no differences in differentiation - efficiency between Controls and the two PMDS patients were present (t-test, n = 3 technical replicates from 2 cell lines per group; *Villin* (CTRL1 vs. CTRL2) p = 0.161; (PMDS1 vs. PMDS2) p = 0.331; (pooled CTRL vs. PMDS) p = 0.8547 (n = 6); *Cdx2* (CTRL1 vs. CTRL2) p = 0.4043; (PMDS1 vs. PMDS2) p = 0.3244; (pooled CTRL vs. PMDS) p = 0.5894 (n = 6)). **(b)** Using a single-tube format, the significant decrease in *ZIP2* and *ZIP4* mRNA expression levels in PMDS patient samples compared to healthy controls was confirmed (t-test, n = 3 technical replicates from 2 cell lines per group; *ZIP2* (CTRL1 vs. CTRL2) p = 0.5352; (PMDS1 vs. PMDS2) p = 0.0237; (pooled CTRL vs. PMDS) p = 0.0033 (n = 6); *ZIP4* (CTRL1 vs. CTRL2) p = 0.1249; (PMDS1 vs. PMDS2) p = 0.5254; (pooled CTRL vs. PMDS) p = 0.043 (n = 6)). (**c**) Quantification of protein expression in lysate obtained from enterocytes. Results show data pooled from 2 controls and 2 PMDS patients. The right panels show individual data points for each measurement and cell line per group. Three technical replicates were performed per patient/control and the expression levels normalized to Actin. A significant reduction of ZIP2 protein levels can be seen in PMDS patient samples and a clear trend for a reduction in ZIP4 (t-test, ZIP2 (CTRL1 vs. CTRL2) p = 0.119; (PMDS1 vs. PMDS2) p = 0.1773; (pooled CTRL vs. PMDS) p = 0.0478 (n = 6); ZIP4 (CTRL1 vs. CTRL2) p = 0.9232; (PMDS1 vs. PMDS2) p = 0.0817; (pooled CTRL vs. PMDS) p = 0.063 (n = 6)).

Differentiated cells from controls and patients were identified as enterocytes by the expression of the marker proteins Sucrase-Isomaltase (SI) and Peptidase 1 (SLC15A1) (Fig. 2c). A detailed analysis of mRNA expression profiles of trace metal homeostasis genes revealed no significant alterations in selected genes of Cu homeostasis (Fig. 2d). The level of ATP7A (ATPase Copper Transporting Alpha), a gene regulating Cu transport from the small intestine into the blood and leading to Cu deficiency if mutated, ATP7B (ATPase Copper Transporting Beta), a gene associated with copper excretion leading to increased copper levels if mutated, and COMMD1 (Copper Metabolism Domain Containing 1), a regulator of copper homeostasis, were not different between Control and PMDS patient cells. Further, no significant alterations in selected genes involved in Fe homeostasis or general divalent metal transporters were seen (Fig. 2e). As expected for PMDS, a significant lower expression level of *SHANK3* was detected in enterocytes (Supplementary Fig. 3). Interestingly, analyzing important Zn homeostasis genes of the metallothionein family, metal-responsive transcription factor (*MTF*) family, zinc transporter family (*SLC30A*), and Zrt- and Irt-like proteins (*SLC39A*) by a broad screening approach, revealed a trend towards increased levels of *MT2A* and a significant reduction in *SLC39A2 (ZIP2*) and *SLC39A4 (ZIP4*) was observed (Fig. 2f). Next we repeated the quantification of mRNA expression in lysate obtained from enterocytes for *ZIP2* and *ZIP4* using a single-tube format instead of a 96 well approach, confirming equally successful differentiation of the patient iPS cells using *Villin*^41^ and *CDX2* (caudal type homeobox 2)^42^ as markers (Fig. 3a). We could verify the significant decrease in *ZIP2* and *ZIP4* mRNA expression levels in PMDS patient samples compared to healthy controls (Fig. 3b). The alterations in ZIP2 and ZIP4 could also be confirmed on protein level (Fig. 3c). Quantification of protein expression in lysate obtained from enterocytes shows a significant reduction of ZIP2 in PMDS. In addition, a reduction of ZIP4 protein in samples from PMDS patients was observed as a clear trend.

**Figure 4:**
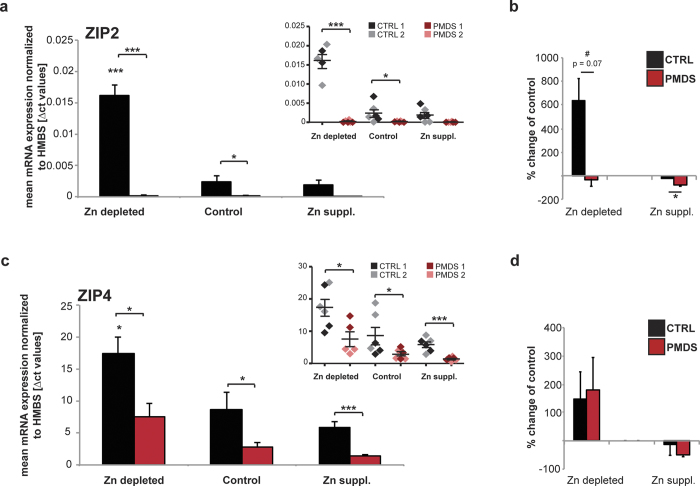
Enterocytes from Controls and PMDS patients were analyzed after exposure to Zn depleted medium and medium supplemented with 10 μM Zn^2+^ for 20 h. mRNA expression is shown as mean Δct values from 3 technical replicates from two controls and two patient cell lines each. (a, c) Smaller inserts (top right of graphs) show individual data points for each measurement and cell line per group. **(a)** Compared to Controls, a significant reduction of ZIP2 expression levels can be found in PMDS patient derived cells under all conditions (2-way ANOVA, main effect of group: F_(1,29)_ = 99.320 p < 0.001; Post hoc analysis Ctrl vs. PMDS: Zn depleted p < 0.0001, Control p = 0.0478, Zn suppl. p = 0.7079. Main effect of treatment: F_(1,29)_ = 47.531 p < 0.001; Post hoc analysis Ctrl vs. PMDS: Zn depleted p < 0.0001. Main effect treatment x group: F_(1,29)_ = 46.340 p < 0.001). **(b)** The increase of ZIP2 expression after exposure to Zn depleted medium, and decrease after exposure to Zn supplemented medium, is significantly altered in PMDS patient enterocytes (two tailed t-test, Ctrl vs. PMDS: Zn depleted p = 0.0787 (indicated as trend “#”), Zn suppl. p = 0.0435). **(c)** Compared to Controls, a significant reduction of ZIP4 expression levels can be found in PMDS patient derived cells under all conditions (2-way ANOVA, main effect of group: F_(1,29)_ = 20.044 p < 0.001; Post hoc analysis Ctrl vs. PMDS: Zn depleted p = 0.0217, Control p = 0.043, Zn suppl. p = 0.0007. Main effect of treatment: F_(1,29)_ = 12.362 p < 0.001; Post hoc analysis Ctrl vs. PMDS: Zn depleted p = 0.042. Main effect treatment x group: F_(1,29)_ = 1.132 p = 0.336). **(d)** The expected response of ZIP4 expression to Zn depleted and Zn supplemented medium is similar in enterocytes from Control and PMDS individuals (two tailed t-test, Ctrl vs. PMDS: Zn depleted p = 0.8468, Zn suppl. p = 0.4489). (**a–d**) Asterisks above bars show significance compared to the control condition, asterisks in between bars compare the two groups (CTRL and PMDS) within a condition.

Expression levels of ZIPs have been reported to respond to local Zn concentrations as part of a regulatory mechanism of Zn homeostasis and uptake. In mice, for example, ZIP4 expression increases under dietary Zn restriction^43^,^44^. We could observe similar regulatory processes in enterocytes derived from healthy individuals (Fig. 4a–d). After exposure to Zn depleted medium, the expression levels of *ZIP2* and *ZIP4* increase significantly in cells from unaffected individuals. Again, we could confirm a significant reduction of *ZIP2* and *ZIP4* expression levels in PMDS patient derived cells under these culture conditions (Fig. 4a,c). In addition, compared to controls, enterocytes from PMDS patients show significantly altered responses to changing Zn concentrations regarding the expression levels of *ZIP2* and *ZIP4*. A significantly reduced up-regulation of *ZIP2* can be seen in PMDS cells and an increased down-regulation after Zn supplementation (Fig. 4b).

### Interaction of SHANK3 and enterocyte Zn transporters in hiPSCs

**Figure 5:**
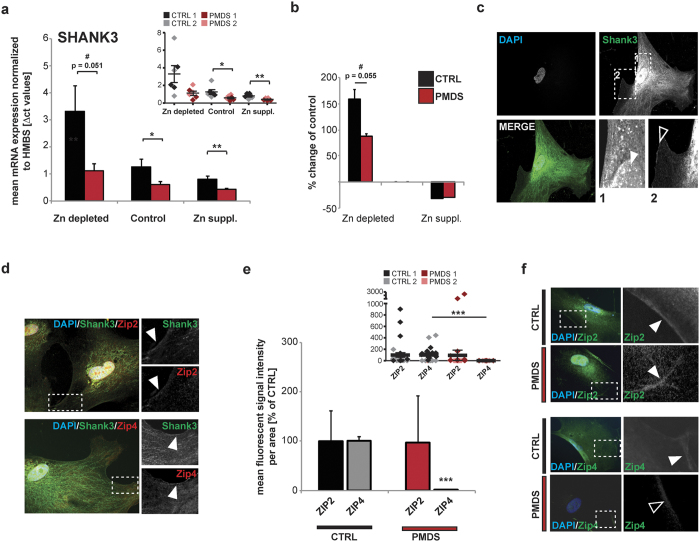
Interaction of SHANK3 and Zn transporters. **(a)** Differentiated enterocytes were analyzed after exposure to Zn depleted and supplemented (with 10 μM Zn^2+^) medium for 20 h. mRNA expression levels are shown as mean Δct values from 3 technical replicates from two controls and two patient cell lines each. Smaller insert (top right of graphs) shows individual data points for each measurement and cell line per group. Compared to Controls, a significant reduction of *SHANK3* expression levels can be found in PMDS patient derived cells under most conditions (2-way ANOVA, main effect of group: F_(1,30)_ = 9.716 p < 0.004; Post hoc analysis Ctrl vs. PMDS: Zn depleted p = 0.051 (indicated as trend #), Control p = 0.0461, Zn suppl. p = 0.0096. Main effect of treatment: F_(1,30)_ = 8.056 p < 0.002; Main effect treatment x group: F_(1,30)_ = 2.589 p = 0.092). **(b)** The *SHANK3* mRNA expression level shows a similar reaction to media with specific Zn concentration as seen for *ZIP2* and *ZIP4*. The up-regulation of *SHANK3* mRNA in Zn depleted medium is significantly less in cells derived from PMDS patients (two tailed t-test, Ctrl vs. PMDS: Zn depleted p = 0.055 (indicated as trend #), Zn suppl. p = 0.9024). **(c)** Immunocytochemistry labeling of SHANK3 reveals cytoplasmic but also nuclear (full arrow) and membrane associated (open arrow) signals of SHANK3. **(d)** Membrane associated SHANK3 co-localizes with ZIP2 (upper panel) and ZIP4 (lower panel) fluorescent signals. **(e)** A significant reduction of ZIP4 signal intensities is seen in PMDS patients compared to controls (t-test, ZIP2 (CTRL1 vs. CTRL2) p = 0.0808; (PMDS1 vs. PMDS2) p = 0.1431; (pooled CTRL vs. PMDS) p = 0.7138 (n = 20); ZIP4 (CTRL1 vs. CTRL2) p = 0.7176; (PMDS1 vs. PMDS2) p = 0.3428; (pooled CTRL vs. PMDS) p = 0.0006 (n = 20)). Smaller insert (top right of graphs) shows individual data points for each measurement and cell line per group. **(f)** For the analysis of signal intensities, cell surface signals were selected and quantified from at least 20 optic fields of view from two patients and two controls.

Using the above mentioned protocol, we found that the expression levels of *SHANK3* that are significantly reduced in PMDS patient derived cells follow a similar expression pattern as seen for *ZIP2* and *ZIP4* (Fig. 5a). However, again, the up-regulation of *SHANK3* mRNA in Zn depleted medium is less prominent in cells derived from PMDS patients (Fig. 5b).

SHANK3, which is localized within the cytoplasm and nucleus, but also at the membranes of enterocytes (Fig. 5c) shows overlapping localization with ZIP2 and ZIP4 (Fig. 5d). Moreover, using immunocytochemistry to quantify signal intensities of cell membrane associated ZIP2 and ZIP4, a significant reduction in ZIP4 was found in cells derived from PMDS patients (Fig. 5e,f). No significant reduction of membrane-associated ZIP2 was measured, pointing towards a reduction of the cytoplasmic rather than the membrane associated pool of the Zn transporter contributing to the observed reduction in overall protein levels (Fig. 5e).

**Figure 6:**
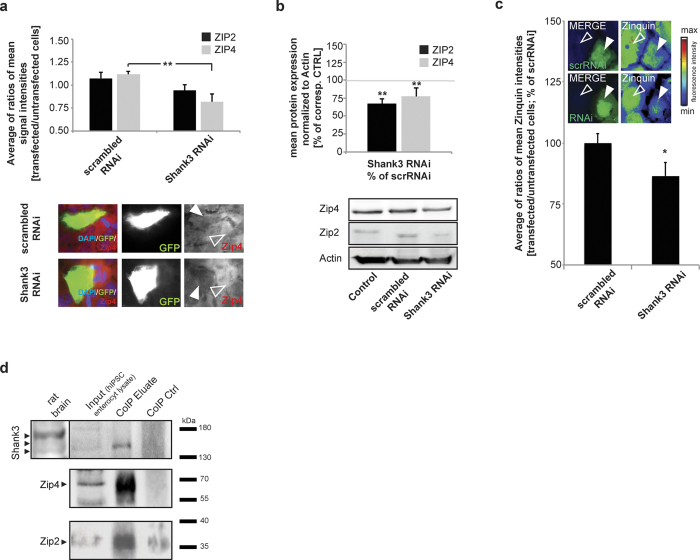
Knockdown of endogenous *SHANK3* in Caco-2 cells. Caco-2 cells were transfected with *SHANK3* shRNA co-expressing *GFP* and the corresponding control, a scrambled shRNA. (**a**) The average cytoplasmic ZIP2 or ZIP4 signal intensity was measured in transfected (full arrow) cells and neighboring untransfected cells (open arrow) and the mean ratio of n = 10 cells is shown. Knockdown of *SHANK3* leads to a significant reduction in ZIP4 signal (*t*-test, p = 0.0015). **(b)** Western blot analysis of protein lysate from Caco-2 cells reveals a significant decrease of ZIP2 and ZIP4 levels normalized to the corresponding control. (n = 3, for statistical analysis, all values were normalized to untransfected controls (Control) and the ratio of scrambled RNAi/Control and *SHANK3* RNAi/Control compared) (*t*-test, ZIP2: p = 0.001; ZIP4: p = 0.0052). **(c)** Caco-2 cells were transfected with scrambled RNAi (scrRNAi) or *SHANK3* specific RNAi for 3 d. 50 μM ZnCl_2_ were added for 30 min and intracellular Zn^2+^ visualized by Zinquin ethyl ester labeling. The Zinquin fluorescence intensity was measured and the ratio between a transfected (full arrow) and untransfected (open arrow) cell calculated. Images for Zinquin show signal intensity color-coded. A significantly (p = 0.0254, *t*-test, n = 10) lower Zinquin signals can be seen in cells expressing the *SHANK3* targeting RNAi compared to cells expressing scrRNAi. **(d)** CoIP experiments using lysate from hiPSC derived enterocytes. All analyzed proteins (SHANK3, ZIP2, ZIP4) were found expressed in enterocytes (Input). Rat brain lysate was used to compare SHANK3 antibody signals in human samples. Anti-SHANK3 antibody was bound to magnetic beads and precipitated. Anti-ZIP4 and anti-ZIP2 were used for detection of possibly co-immunoprecipitated proteins. ZIP4 and to a lesser amount ZIP2 were found co-immunoprecipitated in the analyzed eluate (CoIP Eluate), while no clear band or enrichment was visible in the eluate from a reaction without SHANK3 antibodies coupled to beads (CoIP Ctrl). ZIP2 signals are generally low in lysate from Caco-2 and enterocytes due to low protein expression levels.

As not only SHANK3 can be affected in PMDS, next, to investigate whether alterations in ZIP2 and ZIP4 are specifically dependent on SHANK3 protein levels, we performed overexpression or knockdown experiments of *SHANK3* in Caco-2 cells (Fig. 6a,b; Supplementary Fig 4c–e). Caco-2 cells have been used to study the bioavailability of micronutrients in the past^45^,^46^,^47^ and are a suitable model to study the mechanistic aspects of zinc uptake. Further, Caco-2 cells express SHANK3, ZIP2 and ZIP4. Knockdown of *SHANK3*, which results in approx. 50% reduction of SHANK3 (Supplementary Fig. 4a), leads to a significant reduction of ZIP4 signal intensity (Fig. 6a). For the analysis, the average ratio between signal intensities of transfected and neighboring untransfected cells was used and compared to the corresponding control. In addition, using Western Blot analysis, a reduction of both ZIP2 and ZIP4 was observed (Fig. 6b) under knockdown conditions, while only mRNA levels for *ZIP4* are significantly decreased (Supplementary Fig. 4b). We did not observe increased expression of ZIP2 and ZIP4 after overexpression of *SHANK3* on protein (Supplementary Fig. 4d) or mRNA level (Supplementary Fig. 4e), which could hint towards an upper limit of ZIP expression regulated by other factors.

After treatment of Caco-2 cells with exogenous Zn, cells overexpressing *SHANK3* displayed more nuclear SHANK3 signals and Zn-loaded vesicles with membranes showing SHANK3 signals (Supplementary Fig. 4c). Overexpression of a mutated SHANK3 protein (GFP-SHANK3 R1119X) that has been associated with schizophrenia and that is permanently localized in the nucleus^48^ does not result in a significant increase in ZIP2 and ZIP4 mRNA and protein levels. However, a clear trend towards an increase is visible (Supplementary Fig. 4f,g).

Incubation of Caco-2 cells with ZnCl_2_ solution for 30 min results in a significantly lower fluorescent signal intensity of Zinquin ethyl ester, a membrane permeable Zn fluorophore in *SHANK3* knockdown cells compared to cells expressing scrambled RNAi (Fig. 6c), hinting at a reduced uptake of Zn in *SHANK3* knockdown cells. In Caco-2 cells, across a broad spectrum of Zn levels induced by Zn depletion and supplementation, levels of ZIP2 and ZIP4 assessed by immunocytochemistry are reduced in *SHANK3* knockdown cells compared to cells expressing scrambled RNAi (Supplementary Fig. 5).

Intriguingly, in line with the decrease observed in ZIP4 but not ZIP2 cell surface signal intensity (Fig 5e), using anti-SHANK3 coupled beads to precipitate SHANK3 in lysate from human iPSC derived enterocytes, we primarily found ZIP4 in a complex co-immunoprecipitated with SHANK3 (Fig. 6d). While there was also a somewhat weaker signal for ZIP2, ZIP2 showed some amount of unspecific binding to the beads (CoIP Ctrl).

### Interaction of SHANK3 and enterocyte Zn transporters in mice

**Figure 7:**
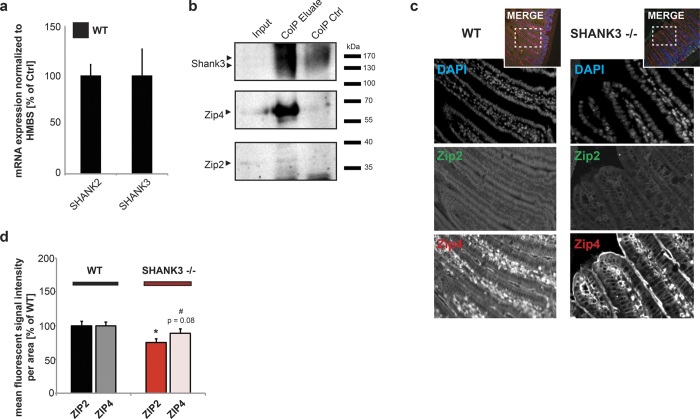
SHANK3 expression and association with ZIP2 and ZIP4 in mice. (**a**) Expression-analysis of SHANK2 and SHANK3 in wildtype mice (n = 3). Both SHANK2 and SHANK3 are expressed in GI epithelium. **(b)** CoIP experiments using lysate from GI epithelium. All analyzed proteins (SHANK3, ZIP2, ZIP4) were found expressed in GI epithelium (Input). SHANK3 was bound to magnetic beads and ZIP4 and ZIP2 were found co-immunoprecipitated in the analyzed eluate (CoIP Eluate), while no clear band or enrichment was visible in the eluate from a reaction without SHANK3 antibodies coupled to beads (CoIP Ctrl). (**c,d**) Immunohistochemistry performed on WT and *Shank3αβ* KO mice (n = 3, 10 weeks of age) reveals a significant reduction in ZIP2 fluorescence intensity and a trend towards a reduction of ZIP4 fluorescence intensity in *Shank3αβ* KO mice compared to WT (t-test, ZIP2: p = 0.01; ZIP4: p = 0.08). For the analysis, fluorescence signal intensity of ZIP2 and ZIP4 positive signals of at least 10 different regions from multiple sections were analyzed.

Similarly to enterocytes differentiated from hiPSCs, SHANK3 was found expressed in the GI epithelium of mice (Fig. 7a). Using co-immunoprecipitation experiments, we found primarily ZIP4 and to a much lesser extend ZIP2 in a complex with SHANK3 in lysate from mouse GI epithelium (Fig. 7b). Moreover, similar to hiPSC, using measurements of ZIP2 and ZIP4 GI sections from WT and *Shank3αβ* KO mice, a significant reduction in ZIP2 fluorescence intensity of the tissue sections and a trend towards a reduction of ZIP4 fluorescence intensity of the tissue sections in *Shank3αβ* KO mice was detected (Fig. 7c,d). These results argue for functional changes *in vivo* such as impaired Zn absorption.

## Discussion

A growing amount of research indicates that abnormalities in the GI system during development might be a factor in ASD. Many patients with ASD have symptoms associated with GI disorders and, especially in young age, show a high prevalence for Zn deficiency. In previous studies, we could show that animals with prenatal zinc deficiency display autism like features^25^,^34^. Further, male mice lacking vesicular zinc in the brain were reported to display ASD like behavior^49^. Therefore, it is possible that the development of ASD is facilitated by Zn deficiency. Of the few studies performed with autistic patients using Zn supplementation, the results show that autistic children with gastro-intestinal co-morbidities significantly improved with respect to hyperactivity and self-stimulatory behavior after Zn therapy^50^. However, detailed studies in human PMDS patients are missing so far.

Here, we investigated whether the observed increased incidence rate of Zn deficiency in ASD also occurs in one of the most prominent syndromic forms of ASD, PMDS, as here, a clear genetic cause is underlying the disorder. Surprisingly, we also found a high incidence rate of Zn deficiency in PMDS patients by analyzing hair samples. At present, about 1000 cases of PMDS are diagnosed worldwide. The prevalence is given as 2.5 to 10 cases per million births^51^. However, it is known from recent studies on large cohorts of autistic patients that the proportion of patients with PMDS lies at about 0.5% within ASD. Thus, the syndrome could be significantly under-diagnosed. Here, we measured hair samples of 31 patients that have clear diagnosis of PMDS and that have not been sampled in our previous study, where Zn levels of 37 participants were determined in blood^25^. Hair and blood Zn values correlate with the advantage of the measurement of hair being more reproducible and sensitive^37^,^52^. Systemic Zn deficiency as assessed in hair or blood samples was published to affect also brain tissue, with up to 30% decreased Zn concentrations in hippocampus and cerebral cortex accompanied by reduced learning behavior in a rat model of diet-induced Zn deficiency^53^,^54^.

Compared to large-scale studies involving hundreds of patients with autism^23^, the sample size of our study is rather small. However, compared to the overall number of diagnosed cases, our two studies cover a wide spectrum of patients located on different continents (North America, Europe, South America). From 68 patients overall combined from both studies, 15 (22.1%) were found to have mild to severe Zn deficiency and the high number of 27 (42.2% based on 64 samples with determination of both Zn and Cu) display low Zn/Cu ratios.

As with Zn deficiency in autism, a major and unresolved question concerns the cause for Zn deficiency. While maternal Zn deficiency during pregnancy might be a major risk factor for autism^26^,^55^, in PMDS and probably also some cases of autism, other factors may be involved. Interestingly, in this study, one Zn deficient patient was treated for his Zn deficiency after the initial hair measurements by the family physician. The patient was supplemented with 10 mg Zn per day for 3 weeks (20 days). After the supplementation, we were able to obtain fresh hair samples and analyzed only few millimeters grown in the period of Zn supplementation. The results imply that Zn absorption is not completely blocked in PMDS and the Zn status can reach normal levels after increased daily Zn uptake. After discontinuation of the supplementation, the patient again developed a Zn deficiency. The potential to compensate a dysregulation of Zn transporters by external factors such as high Zn intake may be a reason why not every patient, but all patient iPSC lines studied show similar molecular alterations. Contrary to defined cell culture media, PMDS patients are exposed to a variety of factors influencing Zn status including food habits and preferences, as well as physical activity.

Several possible reasons for inefficient Zn absorption might be discussed. One of them could be altered expression of Zn transporters and homeostasis genes due to abnormalities in the GI system. In search for such molecular mechanisms that might be responsible for the Zn malabsorption we made use of a cellular *in vitro* system and employed enterocytes generated from iPSC from PMDS patients in comparison to healthy controls. In a comprehensive screen for proteins regulating heavy metal ions in the body we investigated the expression of 27 Zn homeostasis genes. These included the two families of Zn transporters with *ZnT1–8 (SLC30A1-8*) and *ZIP1-14 (SLC39A1-14*). A significant decrease of *ZIP2* and *ZIP4* mRNA levels was found and confirmed in single-tube qRT-PCR. On protein level, for ZIP4, a significant loss of transporters at the membrane occurs and the analysis of cell protein homogenate reveals a strong trend towards a decrease in total ZIP4 and a significant decrease in ZIP2.

Both, ZIP2 and ZIP4 have been shown to regulate Zn uptake into enterocytes. In particular, mammalian ZIP4 is involved in Zn uptake in enterocytes and embryonic visceral endoderm cells. Both, the level of mRNA abundance and subcellular localization of ZIP4 change in response to Zn levels^56^. Mutations in *ZIP4* cause *Acrodermatitis enteropathica (AE)*, a rare and untreated lethal genetic disorder of Zn uptake in humans. Intriguingly, mental problems have been described in *AE* patients such as irritability, a complete lack of smiling and laughing, and an avoidance of eye contact as seen in autistic children^57^. However, dietary Zn supplementation can suppress the symptoms of this disease^58^,^59^,^60^, probably due to the activation of other Zn uptake transporters such as ZIP1-3^61^. The compensatory regulation of ZIP2 was affected in PMDS hiPSCs. Thus, on the level of the individual, one might speculate that in case of exposure to low zinc supply, the decreased expression of ZIP2 and ZIP4 and the altered ability to compensate via regulation of ZIP2 might lead to an increased risk to develop Zn deficiency in PMDS patients.

An interesting observation was the fact that the expression of *ZIP* genes was dependent upon *SHANK3* expression levels. Even though no association has been made so far between control of Zn homeostasis and other deleted genes in the PMDS iPS cell lines used in the study (see Supplementary Table 1), it may have been possible that some have an effect on Zn transporters as well. Therefore, knockdown and overexpression experiments specific for *SHANK3* were performed in Caco-2 cells. Caco-2 cells showed reduced levels of ZIP4 after *SHANK3* knockdown on mRNA level, and protein level assessed by protein biochemistry and immunocytochemistry. Interestingly, after incubation with ZnCl_2_, less uptake of Zn was detected in *SHANK3* knockdown cells underlining the physiological relevance of the reduction of ZIP4 in these cells.

While overexpression does not lead to increased ZIP2 or ZIP4 concentrations, overexpression of a SHANK3 protein with mostly nuclear localization slightly increased both mRNA and protein levels of ZIP2 and ZIP4. Moreover, in PMDS cells, these transporters lost their sensitivity to surrounding Zn concentrations. Further investigations revealed that ZIP4 physically interacts with SHANK3 in enterocytes differentiated from hiPSCs and mouse epithelium, and that primarily ZIP4 can be found in a protein complex with SHANK3.

The data indicate that SHANK3 might be involved in a regulatory pathway for Zn concentrations by direct interaction with Zn transporters at the enterocyte membrane as well as a transcriptional regulator for essential Zn transporters. At the moment it still needs to be elucidated how SHANK3 can mediate a regulatory role on transcriptional events, however, the ability of the protein to localize to the nucleus^48^,^62^ and data presented here might indicate that SHANK3 can not only function as a scaffolding molecule but also as a component of transcriptional complexes. In turn, this would imply that mutations of the *SHANK3* gene can directly cause alterations of transcriptional activity in defined tissues and cell types. A more detailed analysis of surface localization and expression of ZIP2 and ZIP4 in *SHANK3* knockdown and after nuclear localization of SHANK3 should be performed in future studies.

The presence of other “synaptic” proteins in the GI epithelium makes the existence of a protein complex similar to the one described at excitatory postsynapses in enterocytes more than likely. One feature of this complex seems to be the regulation of two Zn importers, *ZIP2* and *ZIP4* by interacting protein networks and possibly nuclear signaling of SHANK3^48^,^62^. Future research will have to investigate whether the deregulation of Zn homeostasis is a common feature of mutations in “synaptic” proteins that are also found in the GI system. The high incidence of trace metal imbalances in patients with autism but also other synaptopathies might hint towards such a common mechanism.

Whenever detected, the treatment of Zn deficiency by oral Zn supplementation is in general advised since persistent Zn deficiency can cause serious health problems including growth impairment, immunological and gastrointestinal problems and neurological symptoms^21^,^63^. Together with the fact that Zn deficiency can contribute to and modify disease symptoms in ASD^26^,^55^, our data implies that Zn therapy should be considered in PMDS and Autism patients as the risk to develop Zn deficiency might be high. Especially, as marginal Zn deficiency in humans often is undetected. Whether Zn therapy also is helpful in absence of Zn deficiency for PMDS or Autism patients with mutation in *SHANK3* needs further investigation and new strategies for Zn delivery bypassing classical Zn uptake pathways may be required.

## Materials and Methods

### Materials

mTeSR1 was purchased from Stem Cell Technologies. RPMI advanced, RPMI 1640, DMEM, DMEM/F12, GlutaMAX, DPBS without Ca^2+/^Mg^2+^, FBS (#10500), Mem Non Essential Amino Acids, Antibiotic-Antimycotic, and TrypsinLE Express were obtained from Gibco. Activin A and FGF-2 were purchased from Cell Guidance Systems and EGF from Invitrogen. Matrigel (hESC qualified) was obtained from BD Biosciences. PBS with Ca^2+/^Mg^2+^ was purchased from PAA. Zinc chloride was obtained from Sigma. Paraformaldehyde was purchased from Merck and D-Saccharose was from Roth. Primary antibodies anti-SI (Sucrase-Isomaltase) (dilution 1:300) and anti-SLC15A1 (Peptidase 1) (dilution 1:300) were purchased from Sigma, anti-SLC39A2 (ZIP2) (dilution 1:10000 ICC; 1:1000 IHC, WB, IP) and anti-SLC39A4 (ZIP4) (dilution 1:200 ICC, IHC; 1:250 WB, IP) from Abcam and Alexa Fluor conjugated secondary antibodies and ProLong^®^ Gold antifade reagent from Invitrogen. For SHANK3 immunoprecipitation and western blotting in-house polyclonal rabbit SHANK3 antibodies were used that have been described previously^25^,^64^ (dilution 1:400). iScript^TM^ cDNA Synthesis Kit, SSoAdvanced Universal SYBR^®^ Green Supermix and customized PrimePCR plates were purchased from Bio-Rad. QuantiTect Primer Assays, RNeasy Mini Kit and QuantiFast^TM^ SYBR_Green RT-PCR kit were purchased from Qiagen. Chelex-100 Resin was obtained from Bio-Rad. CuSO_4_*5H_2_O was obtained from Roth. MgSO_4_*7H_2_O was purchased from Merck. μMACS^TM^ Protein G kit and μMACS^TM^ Separator were purchased from Miltenyi Biotec.

### Analysis of hair zinc and copper in human participants

Patients were enrolled from the “Medical management for children with Phelan McDermid syndrome program” at the Department of Child and Adolescent Psychiatry, Ulm University and the Phelan McDermid syndrome family conference at Wartaweil, Germany. After informed consent of the participants or their legal guardians, a strand of hair was cut close to the scalp at the back of the heads. The hair sample was weighted and after anonymization sent to Torre GmbH (Brandstätterstraße 1, 90513 Zirndorf, Germany) for ICP-MS analysis of zinc and copper. Samples were matched for hair color between both groups. 31 PMDS patients between 1 and 49 years were analyzed (40% female, 60% male). 84% of the patients suffered from infantile hypotonia and 100% of the patients presented speech delay. None of the participants took zinc or mineral supplements. Reference range was 94–306 ppm for zinc and 10–30 ppm for copper. Reference values were calculated as described by Yasuda *et al*.^23^ and compared to reference values initially obtained from healthy normal children and adults^65^ and a study on Vancouver preschoolers aged 2–5 year-old^37^,^66^. The mean Zn level from all participants aged 1–12 was calculated and the obtained standard deviation (SD) multiplied by 2. Values outside 2SD were considered abnormal. The reference range obtained by this method in our study was similar to the range published before^23^.

The patient study was approved by the ethics committee of Ulm University (proposal no. 213/13) and carried out in accordance with the approved guidelines. Hair samples were cut after written informed consent of the study participants or their legal guardians.

### Animals

*Shank3αβ* mutants were generated by Genoway (Lyon, France) and raised on a C57BL/6 background and have been characterized before^64^. All animal experiments were performed in accordance with the guidelines and regulations for the welfare of experimental animals issued by the Federal Government of Germany and by the local ethics committee (Ulm University) ID Number: O.103. The protocol used was approved by the Regierungspräsidium Tübingen, state of Baden-Württemberg, and the ethics committee of Ulm University (ID Number: O.103). Both male wild type and *Shank3αβ* KO mice received the same standard laboratory diet (ssniff GmbH, Germany) with adequate Zn supply and consumed similar amounts of food and water that was accessed *ad libitum*. The housing room was maintained at 22^◦^C, with lights automatically turned on/off in a 12 h rhythm (lights on at 7 am).

### Stem cell-differentiation into enterocyte-like cells

The differentiation was performed using a protocol by Iwao *et al*.^40^. Human induced pluripotent stem cell lines between passages 15 and 20^67^ were grown to 70% confluence in mTeSR1 stem cell medium. Differentiation to definite endoderm was initiated by changing the medium to RPMI advanced supplemented with 2 mM GlutaMAX, 0.5% FBS, 100 ng/ml Activin A and 1% Antibiotic-Antimycotic. After 48 h, cells were grown in RPMI advanced supplemented with 2 mM GlutaMAX, 2% FBS, 100 ng/ml Activin A and 1% Antibiotic-Antimycotic for 24 h. The medium was changed to DMEM/F12 GlutaMAX containing 2% FBS, 250 ng/ml FGF-2 and 1% Antibiotic-Antimycotic for 96 h. FGF-2 is known to drive differentiation from definite endoderm to intestinal stem cells. Subsequently cells were treated with 10 μM ROCK Inhibitor (Ascent Scientific) for 1 h, detached with TrypLE and plated on Matrigel coated 12-well plates. The cells were cultured in DMEM/F12 + GlutaMAX, 2% FBS, 1% NEAA, 2% B27, 1% N2, 20 ng/ml EGF, and 1% Antibiotic-Antimycotic. B27 and N2 were purchased from GIBCO. Medium was changed every 3 days. The cells were harvested after 3 weeks.

### Zn depletion in enterocyte-like cells

For the production of Zn deficient medium, DMEM/F12 + GlutaMAX, 2% FBS, 1% NEAA was depleted of all divalent cations by Chelex-100 (Bio-Rad) purification according to the manufacturer’s protocol as previously described^68^ and subsequently supplemented for all divalent cations present in DMEM/F12 + GlutaMAX except for ZnSO_4_*4H_2_O (1.0505 mM CaCl_2_, 5.2E-6 mM CuSO_4_*5H_2_O, 1.2376E-4 mM Fe(NO_3_)_3_*9H_2_O, 0.0015 mM FeSO_4_*7H_2_O, 0.3005 mM MgCl_2_*6H_2_O, 0.4065 mM MgSO_4_*7H_2_O). After reestablishment of original cation concentrations, the pH of the medium was readjusted with HCl. 2% B27, 1% N2 and 20 ng/ml EGF were added prior to use. Enterocytes were treated for 20 h with either Zn deficient medium, 10 μM ZnCl_2_ supplemented medium or left untreated.

### *SHANK3* Overexpression and Knockdown in Caco-2 colorectal adenocarcinoma cell line

pCMV-MYC (Clontech), MYC tagged *SHANK3* and *SHANK3* RNAi constructs and the SHANK3-R119X mutant construct have been used and characterized previously^68^,^69^. For immunocytochemistry, cells were transfected using the ScreenFect A-plus Transfection Kit (Genaxxon bioscience) according to the manufacturer’s protocol. Cells were imaged or harvested after 3 days. For mRNA analysis and protein biochemistry, Caco-2 cells were transfected with the Nucleofector 4D –X (Lonza, program DG-113) and the SE Cell Line 4D-Nucleofector^®^ X Kit (Lonza) according to the manufacturer’s protocol. 2 × 10^6^ cells and 8 μg DNA were used per cuvette. Three days after transfection, Caco-2 cells were treated with 100 μM TPEN, 50 μM ZnCl_2_ for 30 minutes at 37 °C or left untreated. Transfection efficiency was estimated to 40%.

### Immunocytochemistry

After fixation in 4% Paraformaldehyde/10% Sucrose, the cells were washed with PBS and permeabilized with 0.2% Triton X-100 in PBS for 5 min. Subsequently, blocking solution (5% FBS in PBS) was applied for 1 h. Cells were incubated with the primary antibodies at 4 °C over night. After washing 3x with PBS, the cells were incubated with Alexa Fluor conjugated secondary antibody at room temperature for 1 h. After washing 2x with PBS and 1x with sterile millipore water, cells were mounted with ProLong^®^ Gold antifade reagent with DAPI.

For fluorescent staining of Zn, Zinquin ethyl ester was used. A 5 mM stock solution in DMSO was stored at −20 °C and applied to Caco-2 cells in a concentration of 25 μM for 1 h at RT along with the secondary antibody during immunofluorescence staining.

### Immunohistochemistry

Paraffin embedded sections from intestine were obtained of small intestine from adult mice (10 weeks of age). From each intestine, 4 cm were fixed in 4% buffered formalin. Per sample, three small parts were embedded in paraffin wax longitudinally (for cross sections). After cutting of paraffin embedded sections, (4.5 to 5 μm thick) sections were treated 2x with Xylene for 5 min and 100%, 90%, 70% Ethanol and H_2_O for 5 min each. Subsequently, sections were boiled in 10 mM Sodium Citrat buffer pH 6.0 (Microwave: 15 min 600 W around boiling point). Afterwards the slides were cooled down to room temperature (RT) for approximately 30 min und washed two times in PBS for 2 min each. The tissue on each slide was surrounded with a fat tissue stick. To avoid unspecific antibody binding the tissue was blocked with blocking solution (BS) for 1 h at RT. Subsequently, the tissue was incubated with primary antibody diluted in BS for 2 h at RT in a humid chamber. After washing 3x with PBS for 5 min, the tissue was incubated with secondary antibody, diluted in BS, for 1 h in a humid chamber, followed by a wash step with PBS for 5 min. The tissue was counterstained with DAPI (4′,6-Diamidin-2-phenylindol). Fluorescence images were obtained using an upright Axioscope microscope equipped with a Zeiss CCD camera (16 bits; 1280 × 1024 ppi) using Axiovision software (Zeiss) and analyses of signal intensities of the tissue sections were performed with ImageJ 1.48r.

### Cell lysis and RNA preparation

Cells were harvested and resuspended in 600 μl RIPA buffer, and kept for 30 min on ice. Lysate was cleared from debris by centrifugation (14000 rpm, 15 min) and stored at −80 °C with Protease Inhibitor. Alternatively, lysate from gut epithelium of mice was used. Total RNA was isolated with the RNeasy Mini Kit according to the manufacturer’s protocol. All of the optional purification steps were performed and RNA eluted with sterile RNAse free water.

### qRT-PCR

cDNA synthesis of pooled RNAs was performed with the iScript^TM^ cDNA Synthesis Kit (Bio-Rad) according to the manufacturer’s protocol in a total reaction volume of 20 μl and a maximum of 1 μg RNA/reaction. Quantitative real-time-PCR was performed using the SSoAdvanced Universal SYBR^®^ Green Supermix (Bio-Rad) and customized PrimePCR plates in 96 well format with immobilized primers (Bio-Rad) according to the manufacturer’s protocol with a final reaction volume of 20 μl and 2 ng cDNA/well. Resulting data was analyzed using the hydroxymethylbilane synthase (*HMBS*) or Glyceraldehyde 3-phosphate dehydrogenase (*GAPDH*) gene as an internal standard to normalize transcript levels. Cycle threshold (ct) values were calculated by CFX Manager (version 3.1). All quantitative real-time PCR reactions were run in technical duplicates for each of the 2 patient and control cell lines and mean ct-values for each reaction were taken into account for calculations.

Alternatively, first strand synthesis and quantitative real-time-PCR amplification were performed in a one-step, single-tube format using the QuantiFast^TM^ SYBR_Green RT-PCR kit from Qiagen according to the manufacturer’s protocol in a total volume of 20 μl. Thermal cycling and fluorescent detection were performed using the Rotor-Gene Q real-time PCR machine (model 2-Plex HRM) (Qiagen). The SYBR Green I reporter dye signal was measured. Resulting data were analyzed using the *HMBS* gene as an internal standard to normalize transcript levels. Cycle threshold (ct) values were calculated by the Rotor-Gene Q Software (version 2.0.2). All quantitative real-time PCR reactions were run in technical triplicates for each of the 2 patient and control cell lines and mean ct-values for each reaction were taken into account for calculations.

### Protein biochemistry

To obtain homogenate from GI tissue, small intestinal epithelium was isolated from mesenchyme following a protocol after Nik and Carlsson^70^. In brief, small intestine was cut into 4–5 cm long pieces. Gut mucus is removed by gently squeezing it out of the intestine with the blunt point of tweezers. Each piece was inverted by inserting a rod securing the intestine at one end with a suture and pulling the rod back. The rod with the inverted piece was inserted into a pipet tip (1000 μl) and one end of the intestine pulled onto the tip. After careful removal of the rod, the second end of the intestinal piece is pinched off with a suture. The inverted intestine with the attached pipet tip is submerged in cell recovery solution and repeatedly in- and reflated with air over the course of at least 30 min per piece. During this time the epithelium is separated from the other intestinal layers. For lysis, 2.4 ml of RIPA buffer + PI (Complete EDTA-free Protease Inhibitor Cocktail tablets; Roche, Mannheim) is applied to the collected mouse tissue. To disrupt the epithelium a sonicator is used (4 pulses, lasting 1 s each). Afterwards the lysate is incubated for 2 h at 4 °C on a rotator followed by centrifugation for 20 min at 4 °C at 11700 rpm.

To obtain homogenate from cultures, cells were lysed and homogenized in lysis buffer (150 mM NaCl, 1% Triton X-100, 50 mM Tris–HCl, pH 8.0) containing protease inhibitor (Roche).

Protein concentration was determined by Bradford protein assay. Proteins were separated by SDS-PAGE and blotted onto nitrocellulose membranes (GE Healthcare). Immunoreactivity was visualized using horseradish peroxidase (HRP)-conjugated secondary antibodies and the SuperSignal detection system (Pierce).

For Co-Immunoprecipitation (Co-IP), enterocyte-like cells were washed and detached from the surface using a cell scraper and centrifuged at 3200 rpm. The pellet was lysed in 1.5 ml Triton X Lysis Buffer for 30 min on ice. Alternatively, protein lysate from mouse GI epithelium was used. Immunoprecipitation with 10 μl SHANK3 antibody serum using μMACS Columns and Protein G MicroBeads (Miltenyi Biotec) was performed according to the manufacturer’s protocol.

### Statistic

Statistical analysis was performed using Graph Pad Prism 5 and tested for significance using *t* tests (all values were normally distributed). Two-Way ANOVA with Bonferroni correction was used to identify treatment (Zn depletion/supplementation) or genotype effects, or interaction between the two factors using SPSS version 20. Statistical tests were two tailed with a significance level of α ≤ 0.05. Significances are stated with *p* values < 0.05*; <0.01**; <0.001***. An observed trend (*p* between 0.05 and 0.1) is indicated with “#” and the *p* value stated in the figures.

#### qRT PCR quantification

Relative quantification is based on internal reference genes to determine virtual mRNA levels of target genes. Cycle threshold (ct) values were calculated by the Rotor-Gene Q Software (version 2.0.2). Ct values were transformed into virtual mRNA levels according to the formula: virtual mRNA level = 10 * ((ct_(target)_ − ct_(standart)_)/slope of standard curve).

#### Western blot quantification

Evaluation of bands from Western blots (WBs) was performed using ImageJ. Three independent experiments were performed and blots imaged using a MicroChemi Imaging System from Biostep. The individual bands were selected and the integrated density was measured. All WB bands were normalized to β-Actin or GAPDH and the ratios averaged and tested for significance.

## Additional Information

**How to cite this article:** Pfaender, S. *et al*. Zinc deficiency and low enterocyte zinc transporter expression in human patients with autism related mutations in SHANK3. *Sci. Rep.* **7**, 45190; doi: 10.1038/srep45190 (2017).

**Publisher's note:** Springer Nature remains neutral with regard to jurisdictional claims in published maps and institutional affiliations.

## Supplementary Material

Supplementary Figures and Table
