# Supplementary material for: Zinc deficiency and low enterocyte zinc transporter expression in human patients with autism related mutations in SHANK3
Source: Sci Rep. 2017 Mar 27;7:45190. doi: 10.1038/srep45190 (PMC5366950; doi:10.1038/srep45190)
Supplement: Supplementary Figures and Table [file srep45190-s1.pdf]

Zinc deficiency and low enterocyte zinc transporter expression in human patients with autism related mutations in SHANK3

Stefanie Pfaender, Ann Katrin Sauer, Simone Hagmeyer, Katharina Mangus, Leonhard Linta, Stefan Liebau, Juergen Bockmann, Guillaume Huguet, Thomas Bourgeron, Tobias M. Boeckers, Andreas M. Grabrucker

Supplementary Figures

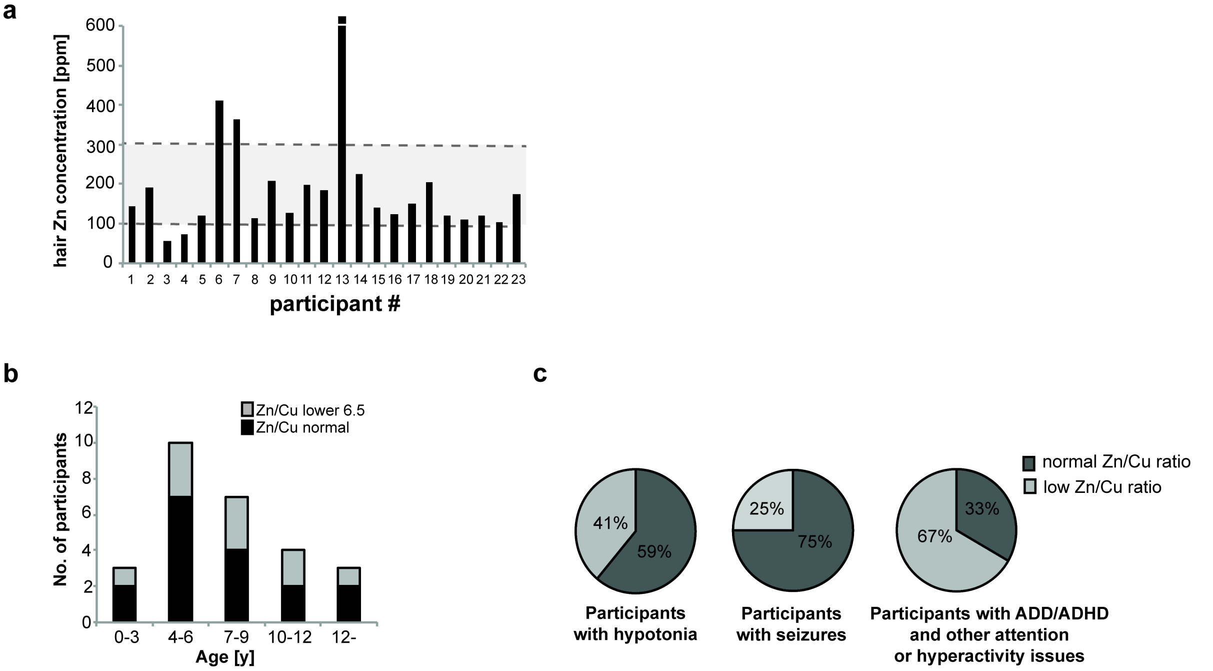

d

		handedness			hypotonia		seizures		ADD/ADHD	
	normal Zn/Cu ratio	5 le	8 ri	4 ?	13	3	6	10	2	13
	low Zn/Cu ratio	3 le	6 ri	1 ?	9	1	2	8	4	6
total		8 le	14 ri	5 ?	22	4	8	18	6	19

		Arachn. cysts		heart issues		skin issues		GI issues	
	normal Zn/Cu ratio	3	13	3	13	3	13	2	14
	low Zn/Cu ratio	0	10	0	10	0	9	2	8
total		3	23	3	23	3	22	4	22

Participant
- with symptom (white cells)
- without symptom (grey cells)

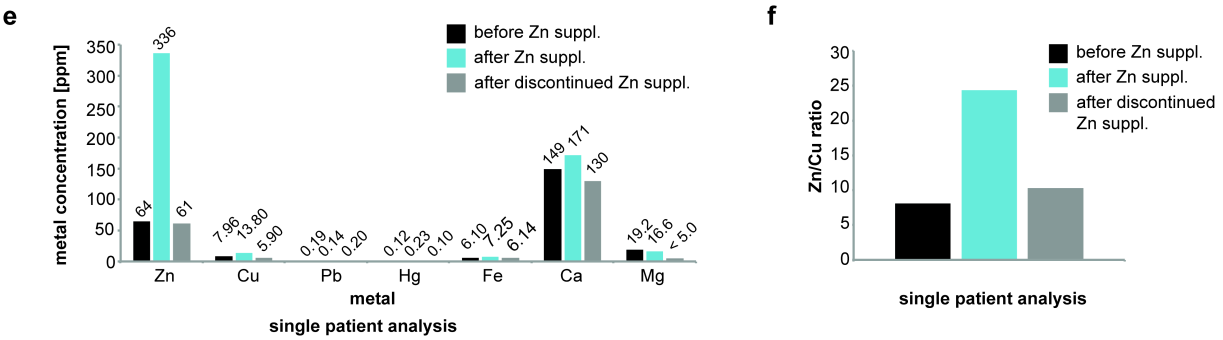

Supplementary Figure 1. Zinc (Zn) and Copper (Cu) concentrations in the hair of participants. **(a)** Hair samples were collected from 23 healthy participants (within the same age range and similar male/female ratio as PMDS patients). Concentrations of Zn were determined using ICP-MS and are shown in ppm. Two participants (8.7%) were identified with Zn levels below the normal range (< 95 ppm). **(b)** Evaluation of patient data from hair analysis of individuals diagnosed with PMDS. The fraction of individuals with low Zn/Cu ratio per age group is shown. Measurements of hair of individuals below three years of age are limited due to limited amount of hair. From five subjects in the group, only in three, Zn and Cu could be measured. **(c,d)** No clear association between low Zn/Cu levels and the presence of hypotonia and seizures in participants was seen. Participants with ADD/ADHD and other attention or hyperactivity issues were frequently found to have low Zn/Cu ratios. **(d)** Due to the limited sample size or limited presence of symptoms, no association between the occurrence of Arachnoidal cysts, and heart and skin problems could be made (not shown). The prevalence of right-handedness seems to be less pronounced in comparison to the general population, with 8 left (le) - handed compared to 14 right (re) - handed participants and 5 participants with no clearly visible side preference. **(e)** One participant (Participant # 21) that displayed low Zn levels was reported to have undergone Zn supplementation with 10 mg Zn per day for 3 weeks and thus was re-evaluated after this period. Only very few millimeters of hair were used to cover the latest period of growth. The results show a strong increase in Zn content. Other trace elements were not severely affected within the period of Zn supplementation. However, several months after discontinued Zn supplementation, a Zn deficiency is re-established. After discontinued supplementation, Mg levels are low, but confirmatory analysis could not be performed based on limited sample size. **(f)** The Zn/Cu ratio that was at the lower limit before supplementation shows a strong increase after Zn supplementation and a drop back after discontinued supplementation.

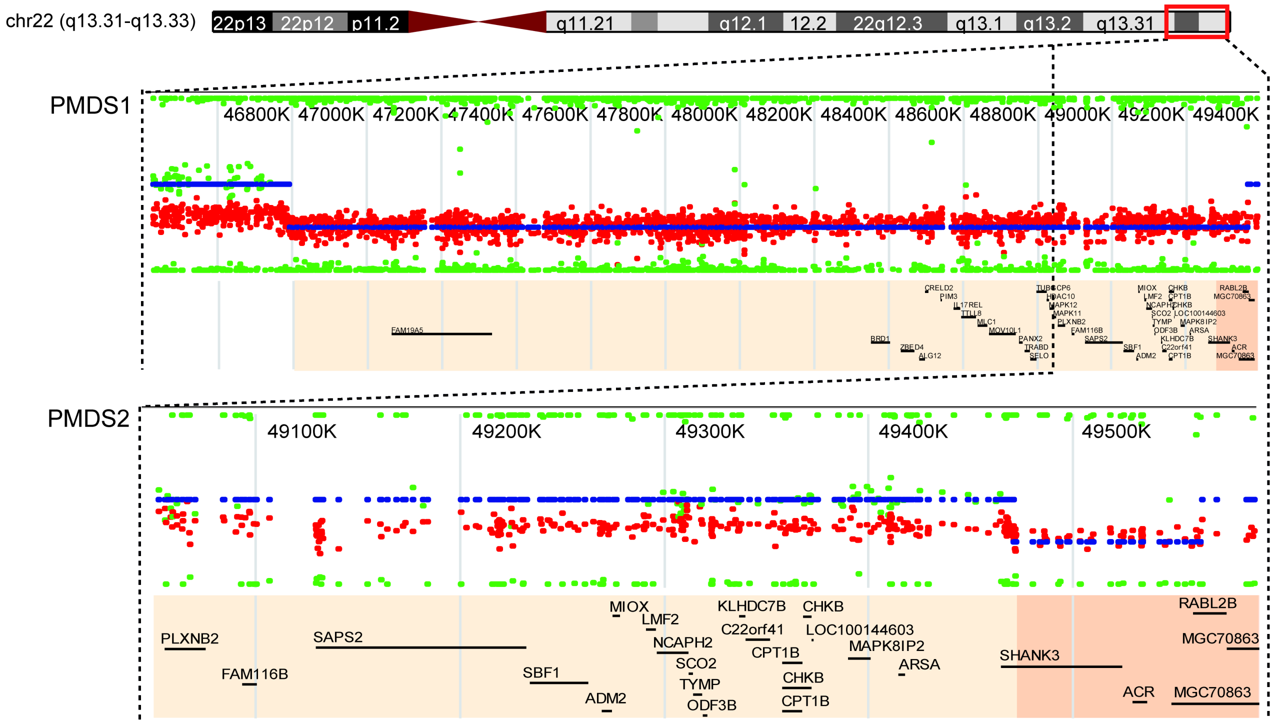

Supplementary Figure 2. Sequencing information from chromosome 22 of PMDS1 and PMDS2. The deletion size and affected genes are shown.

Supplementary Table 1. List of deleted genes on chromosome 22q from patient 1 and 2 and assessment of their putative relevance for Zn homeostasis.

Gene	Name	Function	Relevance for zinc absorption
BRD1	bromodomain containing 1	Component of the MOZ/MORF complex which has a histone H3 acetyltransferase activity. Highly expressed in testis.	no
ZBED4	zinc finger BED-type containing 4	regulation of trasncription, found in human retina	no
ALG12	Alpha-1,6-Mannosyltransferase	member of the glycosyltransferase 22 family.Catalyzes the addition of the eighth mannose residue in an alpha-1,6 linkage onto the dolichol-PP-oligosaccharide precursor. Expressed in fibroblasts.	no
CRELD2	Cysteine Rich With EGF Like Domains 2	may regulate transport of alpha4-beta2 neuronal acetylcholine receptor	unkown
PIM3	Pim-3 Oncogene	Ser/Thr protein kinase. Plays a role in signal transduction cascades related to autophagy, apoptosis, NFkB signaling	unkown
IL17REL	Interleukin 17 Receptor E-Like	integral component of plasma membrane, interleukin-17 receptor activity, unkown function	no
TTLL8	Tubulin Tyrosine Ligase Like 8	protein-glycine ligase activity and sigma factor antagonist activity. Involved in the side-chain initiation step of the glycylation reaction	no
MLC1	megalencephalic leukoencephalopathy with subcortical cysts 1	unknown fucntion, probably a cation channel. Regulates the response of astrocytes to hypo-osmosis by promoting calcium influx.	unkown
MOV10L1	Mov10 RISC Complex RNA Helicase Like 1	encodes a putative RNA helicase and shows testis-specific expression	no
PANX2	Pannexin 2	belongs to the innexin family, structural components of gap junctions	no
TRABD	TraB Domain Containing	unknown function	unkown
SELO	Selenoprotein O	a redox-active mitochondrial selenoprotein, unkown function	no
TUBGCP6	Tubulin Gamma Complex Associated Protein 6	part of a large multisubunit complex required for microtubule nucleation at the centrosome	no
HDAC10	Histone Deacetylase 10	deacetylate lysine residues on the N-terminal part of the core histones	no
MAPK12	mitogen-activated protein kinase 12	signal transducer during differentiation of myoblasts to myotubes. Highly expressed in skeletal muscle and heart.	no
MAPK11	mitogen-activated protein kinase 11	kinase,closely related to p38 MAP kinase, can be activated by proinflammatory cytokines and environmental stress	unkown
PLXNB2	Plexin B2	transmembrane receptor that participates in axon guidance and cell migration in response to semaphorins	no
FAM116B	DENN Domain Containing 6B	Guanine nucleotide exchange factor (GEF) for RAB14 and RAB35	unkown
SAPS2	SAPS Domain Family Member 2	a regulatory subunit for the protein phosphatase-6 catalytic subunit	unkown
SBF1	SET binding factor 1	protein-tyrosine phosphatase. Catalytically inactive, associated with Charcot-Marie-Tooth disease	no
ADM2	Adrenomedullin 2	a member of the calcitonin gene-related peptide (CGRP)/calcitonin family of hormones that play a role in the regulation of cardiovascular homeostasis, prolactin release, anti-diuresis, anti-natriuresis, and regulation of food and water intake	unkown
MIOX	Myo-Inositol Oxygenase	iron ion binding and oxidoreductase activity, acting on NAD(P)H. Kidney specific.	no
LMF2	Lipase Maturation Factor 2	Involved in the maturation of specific proteins in the endoplasmic reticulum. May be required for maturation and transport of active lipoprotein lipase through the secretory pathway	no
NCAPH	Non-SMC Condensin I Complex Subunit H	a regulatory subunit of the condensin complex. This complex is required for the conversion of interphase chromatin into condensed chromosomes	no
SCO2	SCO2 Cytochrome C Oxidase Assembly Protein	a metallochaperone that is involved in the biogenesis of cytochrome c oxidase subunit II	unkown
TYMP	thymidine phosphorylase	promotes angiogenesis and stimulates the growth of a variety of endothelial cells in vitro	no
ODF3B	Outer Dense Fiber Of Sperm Tails 3B	unknown function	unkown
KLHDC7B	Kelch Domain Containing 7B	unknown function	unkown
CPT1B	Carnitine Palmitoyltransferase 1B	a member of the carnitine/choline acetyltransferase family, is the rate-controlling enzyme of the long-chain fatty acid beta-oxidation pathway in muscle mitochondria	no
CHKB	Choline Kinase Beta	catalyzes the phosphorylation of choline/ethanolamine to phosphocholine/phosphoethanolamine	no
MAPK8IP2	mitogen-activated protein kinase 8 interacting protein 2	expressed in brain and pancreatic cells, a scaffold protein involved in c-Jun amino-terminal kinase signaling pathway	no
ARSA	Acrylsulfatase A	hydrolyzes cerebroside sulfate to cerebroside and sulfate. Defects in this gene lead to metachromatic leucodystrophy	no
**SHANK3**	**SH3 and multiple ankyrin repeat domains 3**	**zinc binding multidomain scoffolding protein in synapses, possible role in the nucleus (gene expression)**	**hypothesized here**
RABL2B	RAB, member of RAS oncogene family-like 2B	belongs to the RAS GTPase superfamily, small GTPase mediated signal transduction. Expressed in the testis.	no
ACR	Acrosin	proteinase present in the acrosome of mature spermatozoa.	no

Deleted in PMDS1 (BRD1 to ACR)

Deleted in PMDS2 (MAPK11 to ACR)

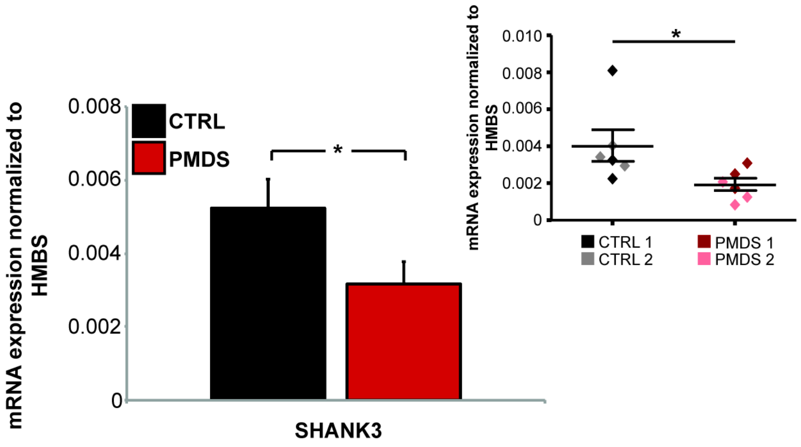

Supplementary Figure 3. Expression analysis of *SHANK* genes in hiPSC derived cells. Expression analysis of *SHANK3* reveals a significant decrease in PMDS patients (n = 2 patients per group analyzed in triplicates). Expression levels are shown normalized to levels of *HMBS*. Smaller insert (top right of graphs) shows individual data points for each measurement and cell line per group (*t*-test, CTRL1 vs. CTRL2: p = 0.5915; PMDS1 vs. PMDS2: p = 0.1197; pooled CTRL vs. PMDS: p = 0.0461 (n = 6)).

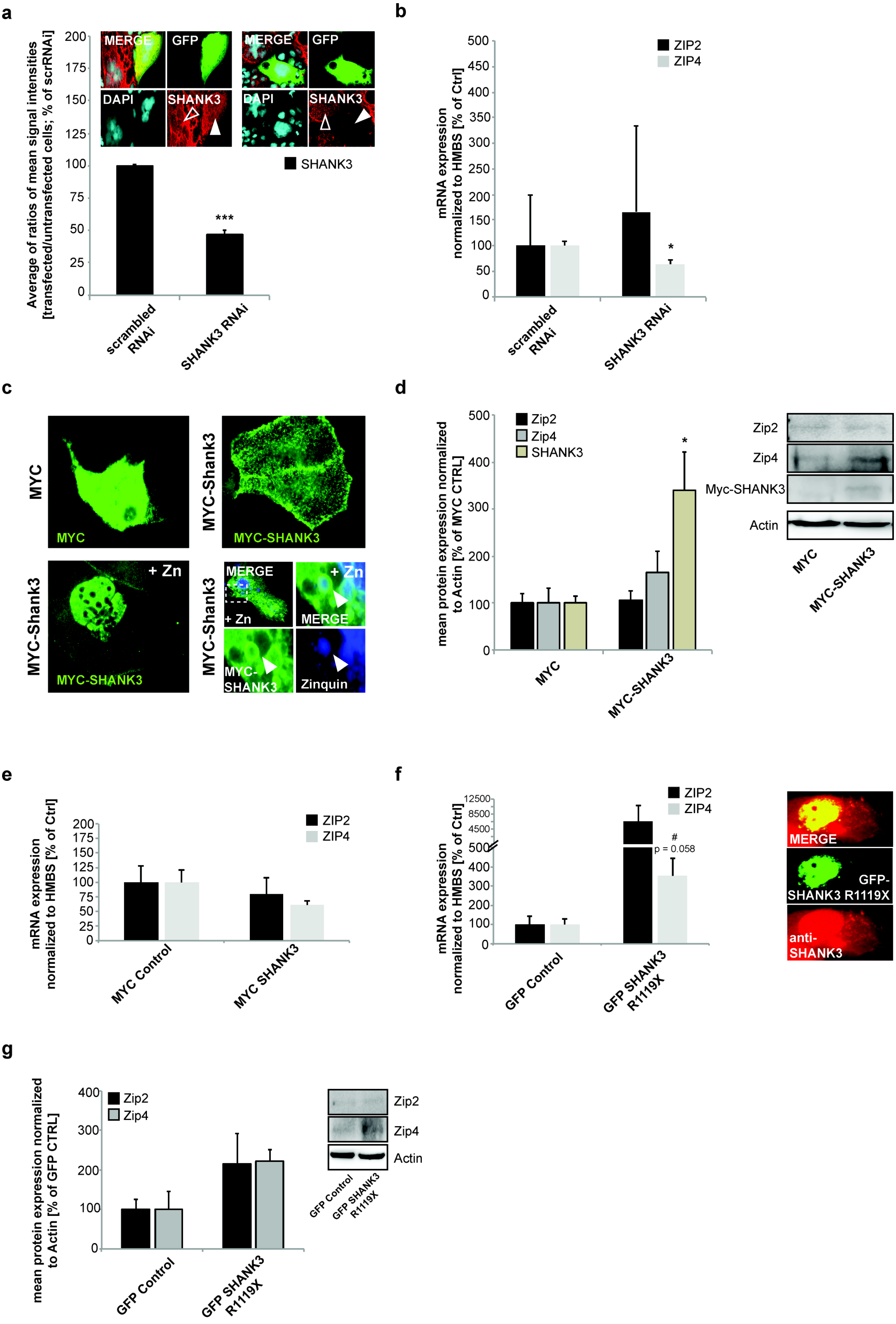
a
b
c
d
e
f
g
MERGE
GFP
DAPI
SHANK3
Average of ratios of mean signal intensities [transfected/untransfected cells; % of scrRNAi]
200
175
150
125
100
75
50
25
0
***
scrambled RNAi
SHANK3 RNAi
mRNA expression normalized to HMBS [% of Ctrl]
500
450
400
350
300
250
200
150
100
50
ZIP2
ZIP4
*
MYC
MYC-Shank3
MYC-SHANK3
+ Zn
MERGE
Zinquin
mean protein expression normalized to Actin [% of MYC CTRL]
Zip2
Zip4
SHANK3
Myc-SHANK3
Actin
MYC Control
MYC SHANK3
12500
8500
4500
#
p = 0.058
GFP Control
GFP SHANK3 R1119X
GFP-SHANK3 R1119X
anti-SHANK3
mean protein expression normalized to Actin [% of GFP CTRL]

Supplementary Figure 4. (a) Caco-2 cells were transfected with scrambled RNAi (scrRNAi) or *SHANK3* specific RNAi and stained for SHANK3. The SHANK3 fluorescence intensity was measured and the ratio between a transfected (full arrow) and untransfected (open arrow) cell calculated. A significant ($p < 0.001$, *t*-test, n = 10) reduction of SHANK3 signals can be seen in cells expressing the *SHANK3* targeting RNAi, corresponding to approx. 50% knock-down. **(b)** Quantification of mRNA expression in lysate obtained from Caco-2 cells transfected with scrambled RNAi or SHANK3 specific RNAi. Results show no significant differences in expression levels of ZIP2 due to generally low expression resulting in a high standard deviation. A significant decrease in ZIP4 mRNA expression is observed in SHANK3 knockdown cells compared to controls (*t*-test, p = 0.0419, n = 3). **(c)** Overexpression of MYC-SHANK3 in Caco-2 cells. SHANK3 proteins cluster at the membrane of Caco-2 cells. After exogenous addition of $ZnCl_2$, increased nuclear staining and staining of Zn-enriched vesicles membranes is seen. **(d)** Western blot analysis of protein lysate reveals a significant increase of SHANK3 levels in SHANK3 overexpressing Caco-2 cultures. No significant alterations in ZIP2 and ZIP4 levels can be observed (n = 3, for statistical analysis, all values were normalized to MYC transfected controls (MYC) (*t*-test, SHANK3: p = 0.0426). **(e)** Quantification of mRNA expression in lysate obtained from MYC and MYC-SHANK3 transfected Caco-2 cells. Results show no significant differences in expression levels of ZIP2 and ZIP4 in SHANK3 overexpressing cells compared to controls (*t*-test, n = 3). **(f)** Quantification of mRNA expression in lysate obtained from GFP and GFP-SHANK3R1119X transfected Caco-2 cells. Results show no significant differences in expression levels of ZIP2 and ZIP4 in SHANK3R1119X overexpressing cells compared to controls. However, a clear trend towards an up-regulation is seen, especially for ZIP4 (*t*-test, ZIP4: p = 0.058, n = 3). **(g)** Western blot analysis of protein lysate obtained from GFP and GFP-SHANK3R1119X transfected Caco-2 cells. No significant alterations in ZIP2 and ZIP4 levels can be observed (*t*-test, n = 3, all values were normalized to controls). A trend towards an up-regulation is seen for ZIP2 and ZIP4.

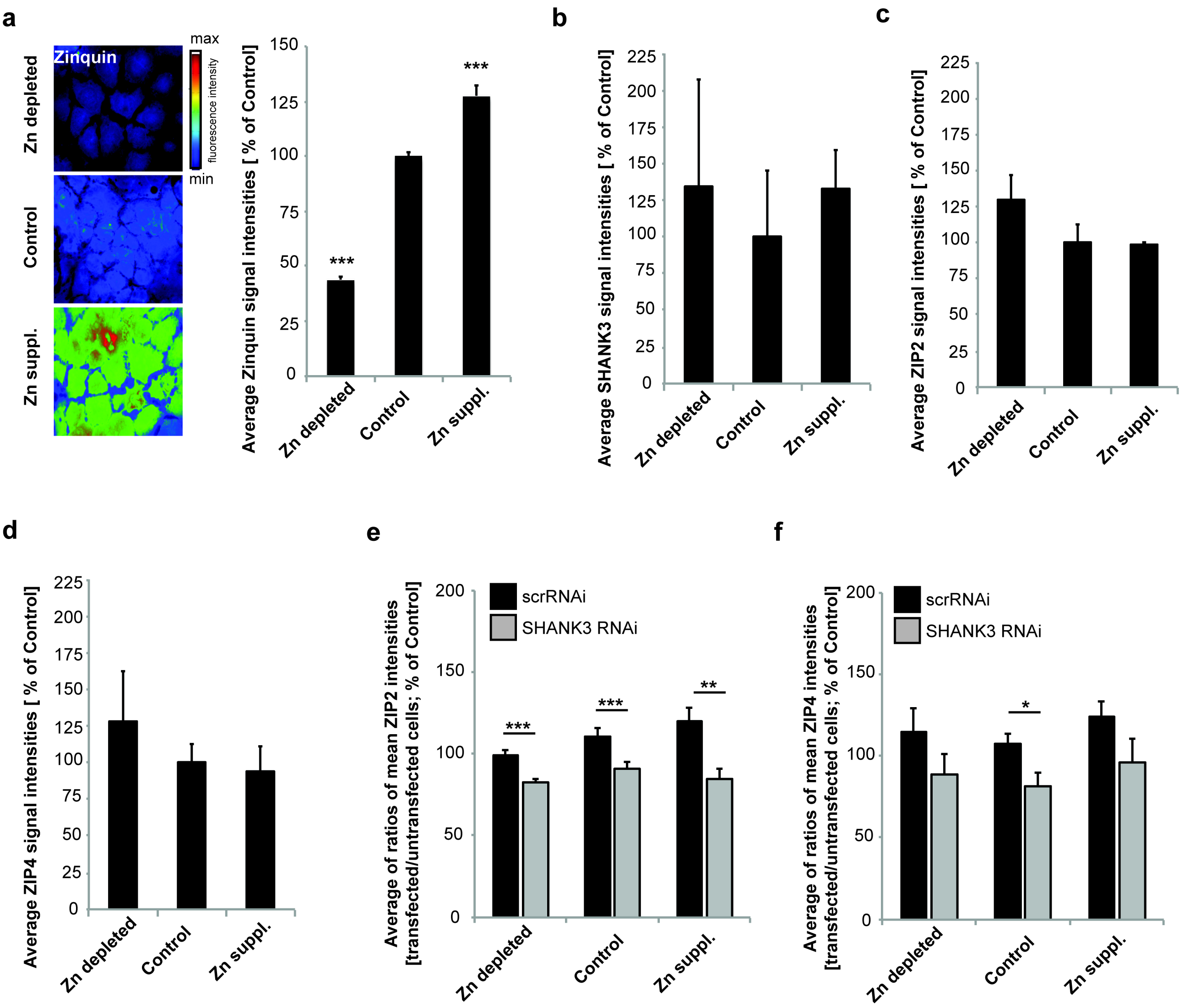

Supplementary Figure 5. (a) Caco-2 cells were treated for 30 min with the Zn chelator TPEN or supplemented with $ZnCl_2$ and compared to untreated cells. Visualization of Zn by Zinquin ethyl ester staining shows a significant decrease of Zn after Zn depletion and an increase after supplementation (one way ANOVA, F = 138.682 p < 0.0001; n = 10; Post hoc analysis shows a significant difference between Zn depleted and Controls p < 0.0001, and Zn supplemented and Controls p < 0.0001). **(b-d)** The signal intensity of SHANK3 **(b)**, ZIP2 **(c)**, and ZIP4 **(d)** was measured under the treatment conditions. No significant differences were detected after 30 min incubation (one way ANOVA, SHANK3: F = 0.219 p = 0.810; ZIP2: F = 1.8863 p = 0.2314; ZIP4: F = 0.587 p = 0.585). A trend towards a compensatory increase in ZIP2 and ZIP4 upon Zn depletion

is visible. **(e,f)** Caco-2 cells were transfected with scrambled RNAi (scrRNAi) or SHANK3 specific RNAi for 3 d and treated for 30 min with the Zn chelator TPEN or supplemented with $ZnCl_2$ and compared to untreated cells. The fluorescence intensity of ZIP2 **(e)** and ZIP4 **(f)** was measured and the ratio between a transfected and untransfected cell calculated. **(e)** Under control conditions, no significant influence of treatment is observed on the immuno-reactive signal intensity of ZIP2 (ANOVA, scrRNAi: $F = 2.423$ $p = 0.108$; SHANK3 RNAi: $F = 0.028$ $p = 0.2972$). However, under all conditions, the levels of ZIP2 are significantly lower in SHANK3 knockdown cells (n = 10, $p_{Zn\ depleted} = 0.0003$; $p_{Control} = 0.0003$; $p_{Zn\ suppl.} = 0.0025$). **(f)** No significant influence of treatment is observed on the immuno-reactive signal intensity of ZIP4 (ANOVA, scrRNAi: $F = 0.781$ $p = 0.468$; SHANK3 RNAi: $F = 0.472$ $p = 0.629$). Under control conditions, the level of ZIP4 is significantly lower in SHANK3 knockdown cells (n = 10, $p = 0.0225$).
